# Transient binocular vision loss and pain insensitivity in Klippel–Feil syndrome: a case report

**DOI:** 10.1186/s13256-024-04374-w

**Published:** 2024-03-06

**Authors:** Zeeshan Ullah, Ayesha Zafar, Hira Ishaq, Zainab Umar, Amir Khan, Yaseen Badar, Nizamud Din, Muhammad Fawad Khan, Pamela McCombe, Nemat Khan

**Affiliations:** 1 Department of Neurology, Lady Reading Hospital, Peshawar, Pakistan; 2 Department of Neonatology, Pakistan Institute of Medical Sciences, Islamabad, Pakistan; 3 Faculty of Medicine, UQ Centre for Clinical Research, Royal Brisbane and Women’s Hospital, The University of Queensland, Herston, Brisbane, QLD 4029 Australia; 4 College of Medicine and Health Science, Khalifa University of Science and Technology, 127788 Abu Dhabi, United Arab Emirates; 5 School of Biomedical Sciences, Faculty of Medicine, The University of Queensland, St. Lucia Campus, Brisbane, Australia

**Keywords:** Case report, Cervical vertebral abnormalities, Congenital insensitivity to pain (CIP), Klippel–Feil syndrome (KFS), Optic disc edema, Rare congenital disorder, Transient vision loss

## Abstract

**Background:**

Klippel–Feil syndrome is a rare congenital bone disorder characterized by an abnormal fusion of two or more cervical spine vertebrae. Individuals with Klippel–Feil syndrome exhibit diverse clinical manifestations, including skeletal irregularities, visual and hearing impairments, orofacial anomalies, and anomalies in various internal organs, such as the heart, kidneys, genitourinary system, and nervous system.

**Case presentation:**

This case report describes a 12-year-old Pashtun female patient who presented with acute bilateral visual loss. The patient had Klippel–Feil syndrome, with the typical clinical triad symptoms of Klippel–Feil syndrome, along with Sprengel’s deformity. She also exhibited generalized hypoalgesia, which had previously resulted in widespread burn-related injuries. Upon examination, bilateral optic disc swelling was observed, but intracranial pressure was found to be normal. Extensive investigations yielded normal results, except for hypocalcemia and low vitamin D levels, while parathyroid function remained within the normal range. Visual acuity improved following 2 months of calcium and vitamin D supplementation, suggesting that the visual loss and optic nerve swelling were attributed to hypocalcemia. Given the normal parathyroid function, it is possible that hypocalcemia resulted from low vitamin D levels, which can occur after severe burn scarring. Furthermore, the patient received a provisional diagnosis of congenital insensitivity to pain on the basis of the detailed medical history and the findings of severe and widespread loss of the ability to perceive painful stimuli, as well as impaired temperature sensation. However, due to limitations in genetic testing, confirmation of the congenital insensitivity to pain diagnosis could not be obtained.

**Conclusion:**

This case highlights a rare presentation of transient binocular vision loss and pain insensitivity in a patient with Klippel–Feil syndrome, emphasizing the importance of considering unusual associations in symptom interpretation.

## Background

Transient binocular vision loss (TBVL) is usually caused by complex migraines, occipital epilepsy, papilledema, or trauma [1]. In contrast, transient monocular vision loss (TMVL) is commonly associated with giant cell arteritis (GCA), retinal artery occlusion, and thromboembolic events [2]. A proper history into the timing, pattern, triggering factors, and accompanying symptoms frequently offers insights into determining the underlying cause of the episode [3]. Klippel–Feil syndrome (KFS), first described by Maurice Klippel and Andre Feil in 1912, is a rare congenital bone disorder distinguished by abnormal fusion of two or more cervical spine vertebrae [4, 5]. Patients with KFS show significant clinical heterogeneity that ranges from skeletal abnormalities, visual and hearing impairment, orofacial anomalies, or visceral anomalies including cardiac, kidney, genitourinary, and neurologic [6]. Herein, we report a case report of a patient with KFS who was affected by TBVL and generalized insensitivity to pain stimuli, which we believe has never been reported for this rare syndrome.

## Case presentation

A 12-year-old Pashtun female presented to our neurology unit for the evaluation of sudden bilateral painless visual loss. There was no history of headache, vomiting, seizures, or trauma. There was also a history of severe sensory impairment—the patient had been badly burned at age 7 years when she caught fire while standing near the stove, with no appreciation of pain until the fire reached the level of the neck. She has been diagnosed with Klippel–Feil Syndrome (KFS) previously due to spinal deformities that were noted after her second birthday by her parents; however, she was clinically assessed, and a definitive diagnosis of type III KFS with Sprengel’s deformity was made at the age of 4 years. There was no significant antenatal history of fever, hypertension, diabetes, or drug abuse. She was delivered vaginally at full term with no delayed cry. There was no delay in developmental milestones. She was assessed to have normal physical and mental development. The patient was one of the seven children of couple from a non-consanguineous marriage. There was no history of developmental abnormalities in her siblings.

On examination, visual acuity was noted as perception of light (POL) in both eyes along with bilateral optic disc edema on fundal examination. Anterior chamber examination was normal. There was a generalized decrease in the sensation of painful stimuli (hypoalgesia) during the pinprick test and thermal test; however, the patient could still localize touch. Vibration and joint position sense were normal. She had intact hearing, taste, and smell. She had extensive scarring of abdomen and chest secondary to burns. Her neck movements were restricted on passive flexion, extension, and tilting. She had winging of scapula bilaterally with Sprengel’s deformity and kyphoscoliosis. The other cranial nerves, speech, autonomic, and motor examination were unremarkable. There were no cerebellar signs.

**Fig. 1:**
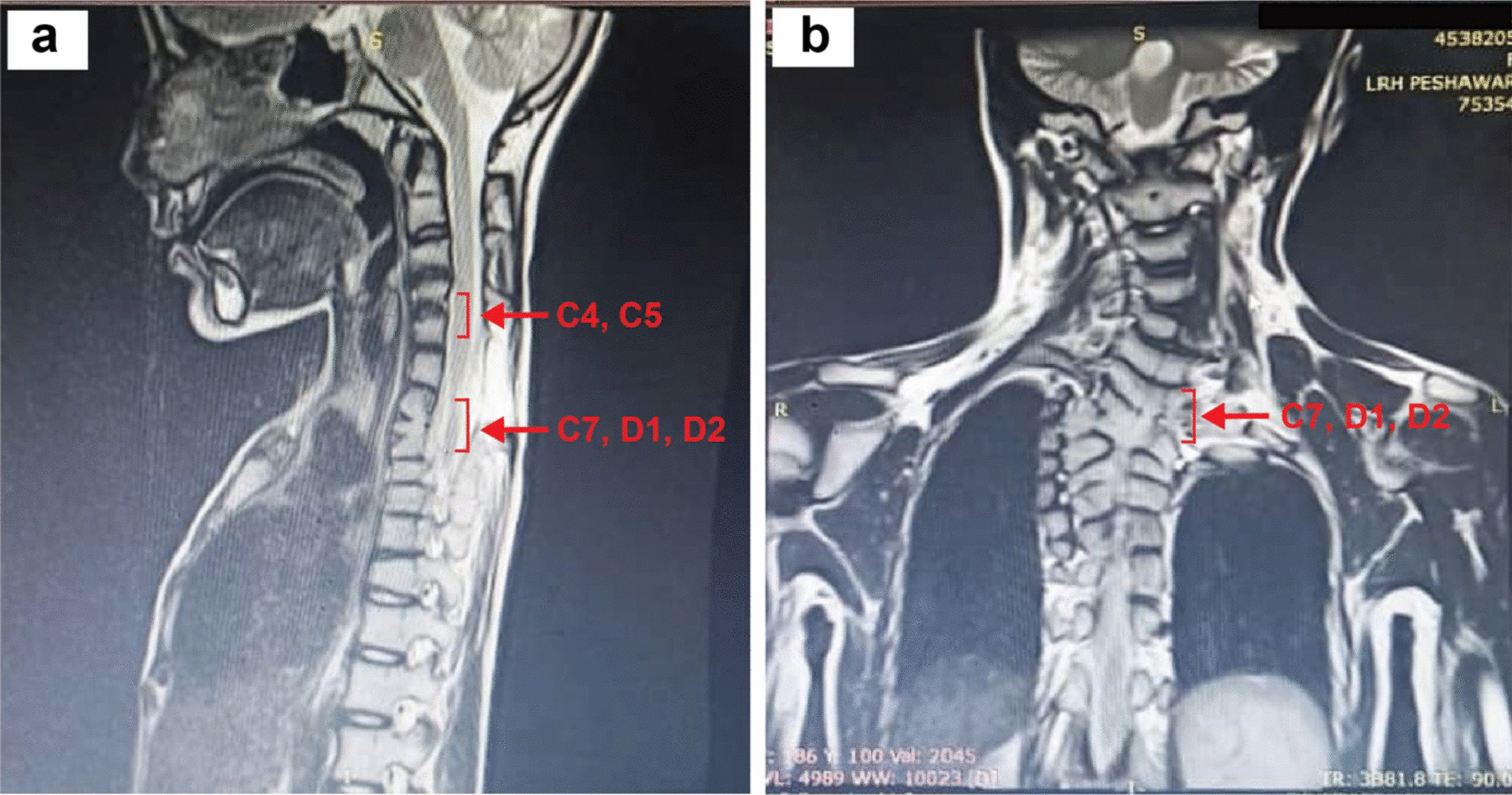
T2-weighted imaging of cervical spine of a 12-year-old female patient born with KFS (Type III). (**A**) Saggital view indicating hemivertebrae at the C4–C5 levels and hemivertebrae with fusion at the C7, D1, and D2 levels. (**B**) Coronal view showing hemivertebrae with fusion at the C7, D1, and D2 levels (red arrows indicating fused vertebrae)

Brain magnetic resonance imaging (MRI) with magnetic resonance venography (MRV) was unremarkable. Cerebrospinal fluid (CSF) pressure, measured by lumbar puncture, was 10 cm of H_2_O. Her hypoalgesia was further confirmed when she did not feel any pain during lumbar puncture. MRI cervical spine revealed hemi-vertebrae at the C4–C5 and D6 levels and hemivertebrae and partial fusion posteriorly at the C7, D1, and D2 levels (Fig. 1). At the C6 level, there was a partial defect in left vertebral lamina with no evidence of bony spur/fibrous septa. It also showed evidence of double cord (split cord malformation). Cervical spine imaging was negative for syrinx. MRI of lumbar spine was consistent with spina bifida occulta at the S1–S3 level with tethered cord (Fig. 1). Nerve conduction study (NCS) was unremarkable and showed no evidence of large fiber neuropathy.

During baseline investigations on the first visit (T0), patient showed microcytic hypochromic anemia with persistent hypocalcemia (Table 1). Her vitamin D level was low, that is, 8.56 ng/ml; however, her serum parathyroid hormone (PTH) was within normal limits. We attributed her TBVL to low serum calcium, which is one of the causes of papilledema after excluding other causes.

**Table 1:** Baseline ﻿investigation of a patient with KFS presented with hypoalgesia and hypocalcemia-induced vision loss

Serial No.	Test	Results (with normal range)
1	Complete blood count	Hb 10.6—microcytic hypochromic Hb electrophoresis was suggestive of thalassemia trait HbA 94%; HbA2 4.7%; HbF 1.1%
2	Renal function tests	Normal
3	Liver function tests	Normal
4	Serum electrolytes	Normal
5	Urine routine exam	Normal
6	Total calcium	6.8 mg/dl (8.5–10.2 mg/dl)
7	Vitamin D	8.56 ng/mL (> 20 ng/mL)
8	Serum PTH	70.9 pg/mL (16–70 pg/mL)
9	Vitamin B_12_	Normal
10	Testosterone	2.5 ng/dl (10–90 ng/dl)
11	LH/FSH	3.56/1.63 IU/ml (pre-puberty, 0–4 IU/ml)
12	Thyroid-stimulating hormone	2.14 mIU/l (0.2–4.5 mIU/l)
13	Serum estradiol	11.80 pg/ml (pre-puberty, 1.6–2.6 pg/ml)
14	CSF pressure	10 cm of H_2_O (7–18 cm of H_2_O)
15	Echo	Normal
16	Ultrasound abdomen/pelvis	Normal
17	MRI brain with MRV	Unremarkable
18	MRI cervical spine	Hemi-vertebrae at the C4–C5 and D6 levels. The C7, D1, and D2 levels showed hemivertebrae along with partial fusion posteriorly. Also, double cord with no evidence of bony spur/fibrous septa at C6 level with partial defect in left vertebral lamina
19	MRI lumbar spine	MRI of lumbar spine was consistent with spina bifida occulta at the S1–S3 level with tethered cord

*CSF* Cerebrospinal fluid; *dL* decilitre; *Echo* echocardiogram; *FSH* follicle stimulating hormone; *IU* international units; *Hb* haemoglobin; *HbA2* hemoglobin subunit alpha 2; *HbF* fetal haemoglobin; *HbA*adult haemoglobin; *LH* luteinizing hormone; *PTH* parathyroid hormone; *cm* centimetre; *m* milli; *pg* picogram; *mg* milligram; *mL*millilitre; *MRI* magnetic resonance imaging; *MRV* Magnetic resonance venography; *ng* nanogram; *TSH* thyroid stimulating hormone

Considering her vision loss to be secondary to hypocalcemia, we commenced oral supplements of calcium and vitamin D. During subsequent visits, her vision gradually improved. Initially, she had perception to light bilaterally on visual acuity assessment. A follow-up visit after 1 month (T1) showed improved visual acuity to 6/24 in the left eye and 6/9 in the right eye. On the second follow-up visit after 2 months (T2), she had 6/9 vision in her left eye and 6/6 vision in the right eye with resolution of papilledema.

## Discussion and conclusions

Our current case report identified a female patient with KFS with unusual presentation of transient binocular vision loss (TBVL) and widespread hypoalgesia. To our knowledge, these anomalies have not been reported previously in patients with KFS.

KFS is a rare congenital abnormality characterized by fusion of at least one pair of adjacent cervical vertebrae, often resulting in a shortened neck, lower hairline, and limited neck mobility [7]. Our patient had all the three features and had evidence of Sprengel’s deformity [6]. Samartzis *et al.* classified KFS into three different types (types I–III) on the basis of the extent of cervical fusion; this classification system is widely used to study the prognosis of such patients [6, 8]. Type I involves single-level congenital fusion of cervical segment; type II comprises multiple, noncontiguous congenitally fused segments; and type III includes multiple, contiguous congenitally fused cervical segments [8]. On the basis of this criterion, our patient was classified as type III patient with KFS. The clinical presentation of KFS is highly heterogenous and can range from asymptomatic to severe, the latter being associated with a number of neurological symptoms [6]. Neurological anomalies associated with KFS include hearing loss, thinning of the corpus callosum, atlanto-occipital subluxation, syringomyelia, hydrocephalus, split cervical spinal cord, meningomyelocele (MCC), and pyramidal tract decussation failure [9, 10]. KFS, having female preponderance, is usually associated with variants in a range of genes, including *GDF6*, *MEOX1*, *GDF3*, *MYO18B*, and *RIPPLY2*, that encode proteins involved in somite development [11]. KFS is also associated with the PAX family of regulatory genes that are implicated in sclerotomal resegmentation that is critical for normal vertebra development [12, 13]. Although the process of cervical vertebra fusion is recognized and used to diagnose patients with KFS, the underlying mechanisms associated with the spectrum of anomalies of KFS are not fully understood [4].

The binocular vision loss in our patient was associated with swollen optic discs and hypocalcemia. Calcium is critical for nerve conduction, and hence hypocalcemia can impair axoplasmic conduction, leading to optic disc edema and vision loss [14]. Hypocalcemia-induced optic nerve head dysfunction can have varying presentations. It may cause true papilledema with raised intracranial pressure or optic disc edema secondary to optic neuropathy with normal intracranial pressure [15, 16]. Similarly visual changes can range from minimal to marked visual loss depending on underlying pathophysiology [15, 16]. It is also well established that parathyroid gland regulates calcium levels in the blood through PTH; hence, papilledema is commonly reported with hypoparathyroidism [17]. However, our patient showed normal serum PTH. Previously, one study has reported hypoparathyroidism in a 72 year old patient with KFS [18].

However, in the present case, parathyroid function was normal, and hypocalcemia was associated with hypovitaminosis D, which was most likely attributable to large body surface area burns sustained by our patient. It has been reported that pediatric burns can cause decreased epidermal synthesis of vitamin D, in scar and adjacent healthy skin, up to 7 years after burn injury [19]. Bilateral disc swelling and vision improved within 2 months in our patient following treatment with calcium and vitamin D supplements. Several cases of hypocalcemia-induced optic disc edema have been reported previously showing improvement with calcium therapy [14, 20].

In the present case, the burns were associated with impaired appreciation of pain. Nociceptive acute pain is critical for normal function, as it provides warning about harmful stimuli [21]. Because the hypoalgesia was severe, it is possible that the subject has congenital insensitivity to pain (CIP), which is an autosomal recessive disorder in which patients are unable to perceive pain but have intact sensation to other stimuli such as vibration, proprioception, and light touch [22]. Such individuals are unable to feel pain in any part of their body, and this situation usually leads to accumulation of injuries that may affect life expectancy [23]. Diagnosis for CIP is primarily clinical, based on impaired pain and temperature perception that is picked up by the family during early childhood. In agreement to this, our patient also showed generalized pain insensitivity that went unnoticed until the subject was affected by severe burns during childhood. Around > 20 genes have been reported to date that can lead to altered neuronal excitability of peripheral nociceptors and cause genetic painlessness [22]. CIP is mostly caused by loss-of-function mutations in SCN9A, gene encoding the protein voltage-gated sodium channel Nav1.7, widely expressed on nociceptors [23]. The *SCN9A* gene mutations result in the production of nonfunctional alpha subunits that cannot be incorporated into Nav1.7 channels and hence impairs the transmission of pain signals from the site of injury to the brain, causing those affected to be insensitive to pain [24]. Other rare genetic mutations associated with CIP include *CLTCL1*, *NGF*, *PRDM12*, *SCN11A* and *ZFHX2* [24].

Other possible diagnosis in our subjects could be another type of sensory neuropathy due to dysfunction of pain-dominant small fibers (Aδ and unmyelinated C fibers) and ataxia-predominant large Aβ fibers [25]. Furthermore, we also ruled out prediabetic condition and vitamin B_12_ deficiency in our patient. Finally, it is possible that the insensitivity to pain was associated with KFS. There was no previous report of insensitivity to pain in KFS. Indeed, one previous report has shown hyperalgesia (increased pain sensitivity) in the extremities and neck of a patient with KFS [26]. However, since KFS is associated with other neurological abnormalities [6], it is possible that sensory loss could be part of the phenotype.

Due to the limited scope of our study, we could not perform genetic testing to confirm the type of mutation for KFS or any neurological channelopathy or neuropathy that could have caused CIP in our patient. Future studies in patients with KFS are warranted to study potential genetic mutation for the voltage-gated sodium channels, Nav1.8 (*SCN10A*) and Nav1.9 (*SCN11A*) in addition to Nav1.7 (*SCN9A*), that could be associated with small-fiber neuropathy and congenital insensitivity to pain [27, 28].

## Conclusion

This case highlights a rare presentation of TBVL and pain insensitivity in a patient with KFS, emphasizing the importance of considering unusual associations in symptom interpretation. Recognition of hypocalcemia-induced papilledema, a rare consequence of KFS, underscores the need for comprehensive evaluation and management. Early intervention, including calcium and vitamin D supplementation, led to a positive outcome, suggesting a potential link between TBVL and metabolic abnormalities in KFS.

## Data Availability

All data underlying the results are available as part of the article. Data was derived directly from the patient’s medical record, and no additional databases were used.

## Abbreviations

CIP: Congenital insensitivity to pain
CSF: Cerebrospinal fluid
FSH: Follicle-stimulating hormone
GCA: Giant cell arteritis
NCS: Nerve conduction study
KFS: Klippel–Feil syndrome
LH: Luteinizing hormone
MMC: Meningomyelocele
MRI: Magnetic resonance image
MRV: Magnetic resonance venogram
POL: Perception to light
PTH: Parathyroid hormone
TMVL: Transient monocular vision loss
TBVL: Transient binocular vision loss
